# Developing Electrospun Ethylcellulose Nanofibrous Webs: An Alternative Approach for Structuring Castor Oil

**DOI:** 10.1021/acsapm.2c01090

**Published:** 2022-09-08

**Authors:** M. Borrego, J. E. Martín-Alfonso, C. Valencia, María del Carmen Sánchez Carrillo, J. M. Franco

**Affiliations:** Department of Chemical Engineering and Materials Science, Campus de “El Carmen”, University of Huelva, Chemical Product and Process Technology Research Center (Pro2TecS), 21071 Huelva, Spain

**Keywords:** ethylcellulose, electrospinning, entanglement network, gel-like dispersion, rheology

## Abstract

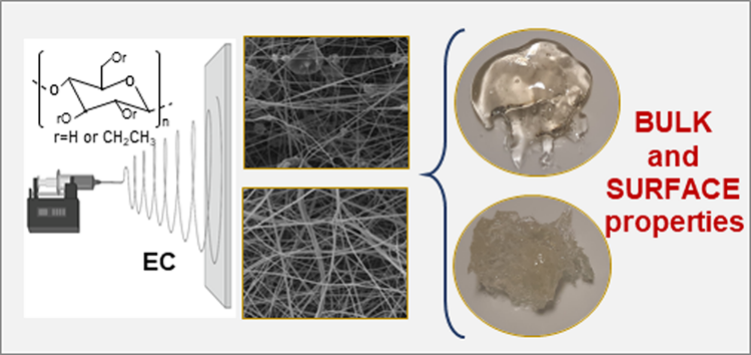

The development of environment-friendly natural polymer gel-like dispersions in oil media, with functional properties, in terms of formulation design and synthesis protocol, is still a cutting-edge research area for many applications. The aim of this work was to study the manufacture of electrospun ethylcellulose (EC) nanofibrous webs and to examine their usage to thicken vegetable oils as an alternative approach. The role of concentration, molecular weight (*M*_w_), and binary solvent systems on the electrospinnability of EC and subsequently on the rheological properties of EC nanofiber dispersions in castor oil was investigated. It was observed that, for each *M*_w_, defect-free nanofibers were produced above a critical concentration, corresponding to about 2.5 the entanglement concentration (*C*_e_). The average fiber diameter increased with both *M*_w_ and EC concentrations. Dielectric constant and dipole moment of binary solvent systems influenced the morphology of the EC nanofiber web. The morphology of the micro- and nanoarchitectures generated played a key role in the physical stabilization and rheological behavior of electrospun EC dispersions. The storage modulus (*G*′) of EC dispersions was correlated with both the spinning solution concentration and average fiber diameter. Furthermore, electrospun EC nanofiber dispersions were compared with EC oleogels obtained by traditional thermogelation from thermorheological and tribological points of view. Overall, this work proposes an efficient and innovative approach to produce bio-based oleogel-like dispersions with great potential in different sectors such as pharmaceuticals, food, or lubricants.

## 1 Introduction

The design and development of electrospun nanofibers and their nanofibrous webs from natural polymers have attracted significant attention over several years to obtain bio-based materials with unique functional properties. These new nanoarchitectures could ideally drive a “smart material” for several engineering applications, such as biotechnology (tissue engineering, controlled/sustained release, etc.), food industry, membranes/filters, textiles, and so on.^1−5^ In particular, cellulose and its derivatives have found profound applications in several engineering fields due to their biocompatibility and biodegradability characteristics.^6^ Being the most abundant polymer on earth, cellulose is widely distributed over a variety of sources, including plants, algae, tunicates, and some bacteria such as *Acetobacter xylinum*.^7^ Cellulose derivatives like methylcellulose, ethylcellulose, cellulose acetate, or carboxymethyl cellulose could be used to design functional nanofibers to provide a feasible approach to produce nanostructured porous materials with promising functionalities, flexibility, renewability, and biodegradability.^8,9^ Ethylcellulose (EC) is a nonionic linear polysaccharide chemically derived from cellulose by ethylation. EC is composed of cellulose backbones with partial substitution of hydrogen hydroxyl end groups by ethyl groups,^10^ and due to its nontoxicity, high flexibility, thermoplasticity, and film-forming ability, it is widely used in food, pharmaceutical, and biomedical applications.^11^ In recent times, the development of functional nanocomposites of cellulose derivatives, and particularity from ethylcellulose, for different applications has drawn attention within the research community.^12−14^ However, the potential of ethylcellulose to develop nanofibers with desired architectures via electrohydrodynamic processing has not been sufficiently explored. Electrospinning is a handy and cost-effective technique that uses an electric field to distort a droplet of a polymeric solution by inducing repulsion between the polymeric chains, thus overcoming the surface tension and allowing a jet to be formed while the solvent is evaporated,^15^ eventually resulting in the formation of polymer fibers with diameters ranging from 2 nm to several micrometers using polymer solutions of both natural and synthetic polymers. This process offers unique capabilities to produce natural nanofibers with controllable pore structures.^16^ Depending on the solution properties, mainly surface tension, rheology, and electrical conductivity, influenced by polymer concentration, and operating parameters (voltage, distance between the tip and collector, flow rate, ambient humidity, and temperature), either electrospinning or electrospraying will occur, thereby producing fibers or particles, respectively, with different architectures. Electrospinning is able to produce a range of nanostructures with different shapes and sizes; particles, beaded fibers, smooth fibers, and ribbons, depending on both solution physicochemical properties and operating parameters, make them favorable candidates to be used in a wide range of applications. The electrospinnability of ethylcellulose has been addressed in a few previous works.^17−19^ Wu et al. discussed the effect of a mixed solvent of tetrahydrofuran (THF)/dimethylacetamide (DMAc) on the surface morphology and diameter distribution of ethylcellulose fibers and found that, using the binary solvent, the diameter of the fibers was thinner and the diameter distribution was narrower than when using either of the two solvents.^17^ Park et al. studied the morphological and surface changes of electrospun EC fibers when using various solvents (THF and DMAc) and their ratios. Regular holes were formed on the surface of the fiber from pure THF and 80 wt % THF in DMAc, while a smooth surface was observed for the pure DMAc and 80:20 wt % DMAc ratio in THF.^18^ Recently, Crabbe-Mann et al. investigated the influence of the EC concentration in ethanol/water at 80:20 (v/v) solutions on their electrospinnability and found that the morphological changes from particles with tails to thick fibers were charted from 17 to 25 wt % solutions.^19^ These results indicated that the solvent type and biopolymer concentration remarkably affected the electrospinning process and the morphology of generated nanofibers.

On the other hand, oil structuring using sustainable thickeners has attracted great interest in both the industry and the academia in recent years, not only in food applications as a promising strategy for fat replacement^20^ but also in the field of pharmaceuticals^21^ and lubricants.^22^ Oleogels are soft materials, consisting of a single self-assembling structuring agent, called gelator, or a combination of different thickener molecules able to form an entanglement network, which traps the oil into its micro- and/or nanostructure. The mechanical and rheological properties of oleogels depend on the interactions among their components (gelators, oils, additives, etc.) and the gelation mechanisms. Only a few biopolymers are able to gel oils by the formation of supramolecular structures through physical entanglements or chemical cross-linking among polymer chains.^23,24^ Among them, EC is considered the only direct oil gelator to date able to generate physically viscoelastic gels in edible oils. The physical gelation of oils with EC is achieved by increasing the polymer/oil mixture temperature above the biopolymer glass transition (∼140 °C) and subsequently cooling down to room temperature, the so-called thermogelation mechanism. EC-based oleogels have shown potential in many applications such as the replacement of fats in foods,^25^ heat resistance agents in chocolates,^26^ oil binding agents in bakery products,^27^ and as the basis for cosmetic pastes.^28^ As previously mentioned, the strategy pursued for oil structuring, in all of these cases, involves a thermogelation mechanism. Besides, other indirect pathways to gel oils using hydrophilic biopolymers have been proposed, such as the so-called foam-templated^29^ and emulsion-templated^30,31^ approaches or stepwise solvent-exchange routes,^32,33^ resulting in porous structures where oil can be adsorbed or entrapped. However, these procedures do not allow us to totally control or tune the self-assembled network formed by EC. Hence, the search for other more suitable approaches to incorporate EC nanostructures into an oil phase, aiming to create tailor-made three-dimensional networks that can likely give rise to oleogels with tunable functional properties, could be ideally welcomed for many applications. In this context, this work proposes an alternative approach to develop physically stable gel-like dispersions based on electrospun ethylcellulose nanofibrous webs and castor oil. Taking into account these considerations, the specific objectives of this work were (i) to study the role of the biopolymer concentration, molecular weight, and binary solvent systems on the electrospinnability of ethylcellulose solutions and (ii) to explore the ability of the different micro- and nanoarchitectures generated to structure castor oil by analyzing the rheological and tribological properties.

## 2 Experimental Section

### 2.1 Materials

Three commercially available ethylcellulose samples (48% ethoxy content) with different viscosity values (EC_1_: 45 cP, EC_2_: 100 cP, EC_3_: 300 cP) purchased from Merck Sigma-Aldrich were used as spinning polymers as received. The higher the EC viscosity, the higher the molecular weight (*M*_w_) is. Hence, *M*_w_ of the different EC samples were 3.9 × 10^4^ (EC_1_), 6.9 × 10^4^ (EC_2_), and 8.2 × 10^4^ (EC_3_) g/mol, and polydispersity indices were as follows: 1.60, 1.15, and 1.09.^34^ The solvents used were acetone (Ac, purity 99.5%), dimethylformamide (DMF, purity 99.8%), dimethylacetamide (DMAc, purity 99.8%), tetrahydrofuran (THF, purity 99.0%), and acetic acid (AA, purity ≥99%). All solvents were supplied by Sigma-Aldrich and used as received without any purification. The main physical properties of these solvents are collected in Table S1 of the Supporting Information. Castor oil (viscosity: 0.55 Pa s, density: 0.958 g/mL, at 25 °C, Guinama, Spain) was stored at room temperature (23 °C), in a dark area, and used as vegetable oil to prepare different dispersions.

### 2.2 Preparation of Spinning Solutions and Rheological Characterization

The influence of EC concentration and molecular weight was investigated using a 1:1 THF/DMAc solvent system. EC was dissolved, at 40 °C, under magnetic stirring (300 rpm) for 2 h, to obtain solutions with concentrations ranging from 1.5 to 14 wt %. Then, the role of solvent systems was then studied for the most promising EC concentration. Homogeneous polymer solutions were prepared by dissolving EC_2_ in different solvent systems: 1:1 THF/DMF, 1:1 THF/DMAc, 1:2 acetone/DMF, 1:2 acetone/DMAc, and acetic acid using the same protocol. All samples were centrifuged for 10 min at 3000 rpm and filtered to check that there were not solids in the solution. Viscous flow measurements of EC solutions were carried out at 23 °C, using an ARES controlled-strain rheometer (Rheometric Scientific, U.K.) equipped with a Couette geometry (internal radius 16 mm, external radius 17 mm, cylinder length 33.35 mm), in a shear rate range of 1–500 s^–1^. At least two replicates were performed on fresh samples.

### 2.3 Electrospinning of EC Solutions and Morphological Characterization

The electrospinning device was constructed using a high-voltage power supply (Spellman High Voltage Electronics Corporation), a syringe with a blunt metal needle, a syringe pump (KD Scientific Pump Company), and a grounded aluminum foil collector. The EC solution was fed through the needle tip by a syringe pump at a flow rate of 0.8 mL/h (tip diameter ≈ 0.6 mm). Nanostructures were produced at a tip-to-target distance of 12 cm and an applied voltage of 20 kV. All of the electrospinning experiments were carried out at 23 °C with a humidity of 45%. The morphology of the electrospun fiber webs was examined using a scanning electron microscopy (FlexSEM 1000 II, Hitachi, Japan) after sputtering the samples with gold under vacuum. All SEM experiments were carried out at an accelerating voltage of 10 kV.

### 2.4 Manufacture of Dispersions of EC Electrospun Nanofiber Webs in Castor Oil and Their Rheological and Tribological Characterization

Electrospun EC nanofibrous webs were dispersed in castor oil, once placed in an open vessel, using an RW-20 mixer by IKA (Germany) coupled with an anchor impeller geometry. Selected electrospun EC nanostructures were dispersed at 15–20 wt % concentrations at room temperature (∼23 °C) for 24 h (see Scheme S1 in the Supporting Information). This procedure involves two steps: first, ethylcellulose nanostructures are obtained via electrospinning and then the nanostructures are dispersed in the castor oil medium to promote the formation of a three-dimensional network. For comparison purposes, EC oleogels were prepared using a magnetic hot plate at a temperature of 150 °C under continuous stirring at 100 rpm until a homogeneous solution was achieved (∼4 h). Samples were rheologically characterized with a controlled-stress rheometer Physica MCR-501 (Anton Paar, Austria) using a serrated parallel plate to avoid possible slip phenomena (25 mm diameter; 1 mm gap). A variety of rheological measurements were performed including steady-state flow tests and small-amplitude oscillatory shear (SAOS) measurements like amplitude, frequency, and temperature sweeps. All measurements were done under isothermal conditions (23 °C), except for obviously temperature ramps. The steady shear flow tests were performed by applying stepped shear rate ramps from 10^–2^ to 100 s^–1^. A long enough data acquisition time was established, at each shear rate, to ensure the achievement of steady-state flow conditions. Frequency sweeps to determine the mechanical spectra were carried out over a 3 × 10^–2^–10^2^ rad/s frequency range, and temperature sweep tests were performed at 0.628 rad/s and a heating rate of 5 °C/min from 25 to 125 °C. Both temperature and frequency sweep tests were performed within the linear viscoelastic regime. The friction coefficient of electrospun EC dispersions and oleogels was measured in a ball-on-three-plates tribological cell coupled with the same rheometer. The three lower plates (made from 1.4301 steel) were fixed through the sample holder forming 45° to the rotating axis, and the upper ball (made from 1.4401 steel) was placed on them with a preset normal load applied. The friction coefficient was obtained by applying a normal load of 30 N and a constant rotational speed of 50 rpm during 600 s at 23 °C. This test was repeated four times to obtain an accurate average friction coefficient.

## 3 Results and Discussion

### 3.1 Electrospinnability of Ethylcellulose Solutions

#### 3.1.1 Effect of Ethylcellulose Concentration on Electrospinning

**Figure 1:**
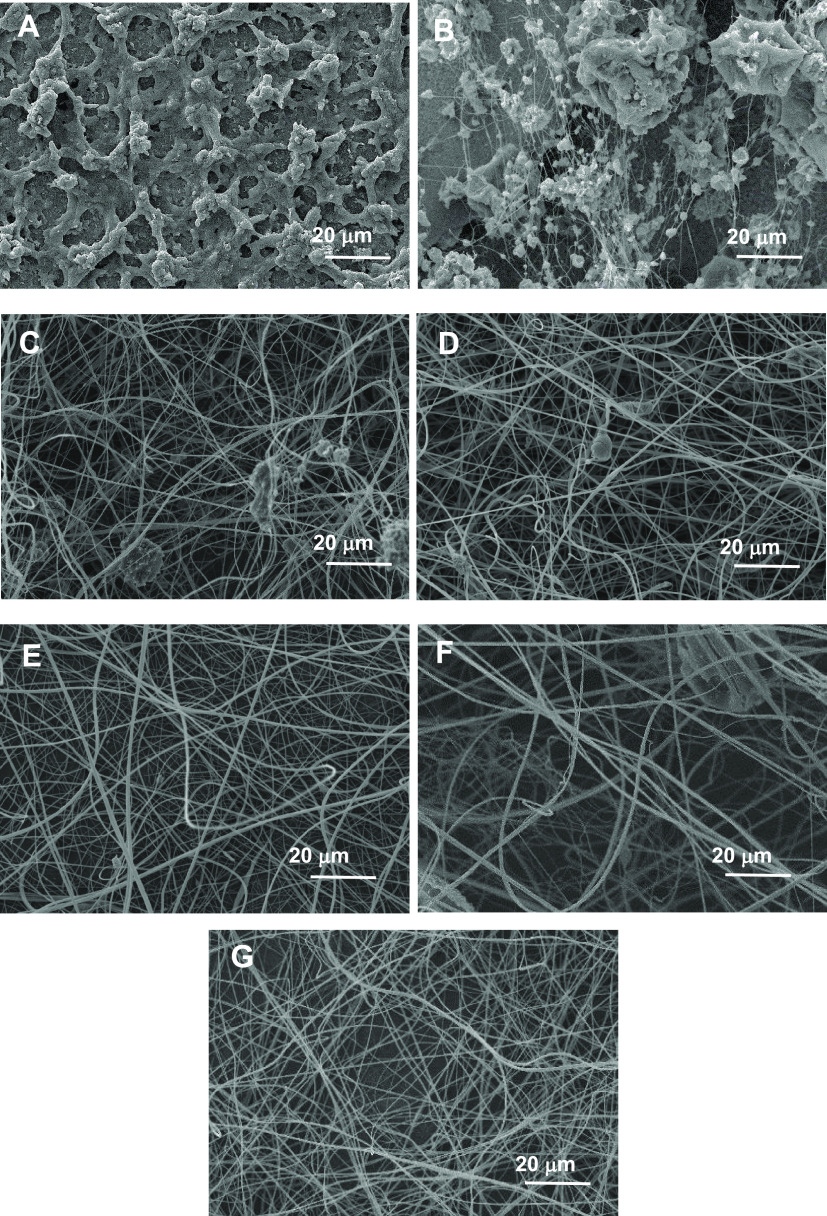
SEM micrographs of electrospun nanostructures obtained from ethylcellulose (EC_2_) solutions in 1:1 THF/DMAc at different concentrations: (A) 2 wt %, (B) 4 wt %, (C) 6 wt %, (D) 8 wt %, (E) 10 wt %, (F) 12 wt %, and (G) 14 wt %.

**Figure 2:**
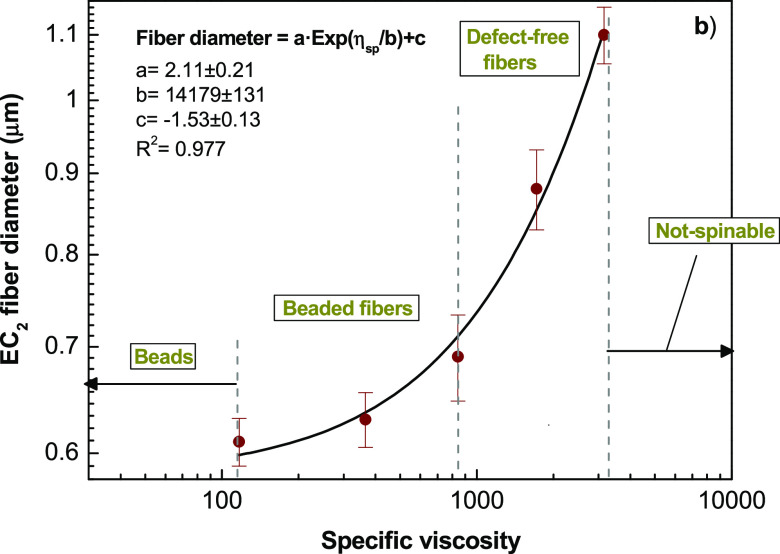
Effect of the specific viscosity of EC_2_ solutions on the electrospun average fiber diameter and electrospinnability.

To study the influence of the solution concentration on nanostructure morphology, EC_2_ (100 cP) solutions in 1:1 THF/DMAc were prepared with concentrations in the range of 1.5–14 wt %. Figure 1 displays SEM micrographs of the EC_2_ electrospun fiber mats collected using different biopolymer concentrations. The results show that not a really fibrous nanostructure but a kind of network composed of clusters of particles was obtained with the 2 wt % EC_2_ solution. The particle formation indicates insufficient chain entanglements. Hence, the study was not extended to concentrations below 2 wt % EC_2_. On increasing the biopolymer concentration to 4 wt %, nanofibers were produced to some extent although many defects and particles were still present. Above 8 wt %, defect-free fibers could be collected. The change in morphology with increasing concentration must be attributed to a competition between surface tension and viscosity. It is well known that to obtain uniform ejection of the charged jet in electrospinning, a solution with a minimum level of polymer concentration is required to exceed an extensive molecular entanglement like a prerequisite for the formation of a stable and continuous jet. This concentration is called the critical entanglement concentration (*C*_e_). If the viscosity of the solution is too low, a continuous stream of the charged jet cannot be formed, as the charged jet experiences instability leading to the formation of droplets; this is called electrospraying.^35^ Conversely, if the viscosity of the solution is too high, the continuous flow of the polymer liquid from the nozzle tip will be prevented. As a result, there is a processing window in terms of the concentration range within which polymer solutions are electrospinnable and beyond that discrete particles are likely to be formed. The critical entanglement concentration, *C*_e_, defines the transition from the semidilute nonentangled to the semidilute entangled regimes. To determine *C*_e_, viscous flow tests of EC_2_ solutions were carried out. All of the systems exhibited a Newtonian behavior, and, obviously, the viscosity increased when the EC_2_ concentration increased from 0.010 to 29.7 Pa·s in the 1.5–12 wt % concentration range, as can be observed in the Supporting Information (Figure S1). The concentration dependence of the specific viscosity (η_sp_=(η_p_ – η_sol_)/η_sol_, where η_p_ is the polymer solution viscosity and η_sol_ is the solvent viscosity) for EC_2_ solutions in 1:1 THF/DMAc is also displayed in the Supporting Information (Figure S2). According to the method proposed by Colby et al.,^36^ which involves the quantification of the concentration dependence of the specific viscosity of linear polymer solutions in good solvents, there are several different (power-law) scaling factors related to the dependences of the specific viscosity upon the polymer concentration in the different concentration regimes. For neutral linear polymers in a good solvent, the specific viscosity is represented as η_sp_ ∼ *C*^1.25^ in the semidilute nonentangled regime and as η_sp_ ∼ *C*^3.75^ in the semidilute entangled domain. For solutions below 3 wt % EC_2_, it was found that η_sp_ ∼ *C*^1.3^, which is consistent with the theoretical prediction for semidilute nonentangled solutions. Above 4 wt % EC_2_, η_sp_ ∼ *C*^3.5^, characteristic of semidilute entangled solutions. The change in slope occurs between 3 and 4 wt % (marked at *C*_e_ ≈ 3.4), which delimits both regimes. As shown by the SEM micrographs in Figure 1, semidilute nonentangled solutions produce clusters of particles, while semidilute entangled solutions produce fibers with increased uniformity when qualitatively compared to nonentangled solutions; concentrations above 8 wt % EC_2_ produces totally uniform fibers. The solution at a 4 wt % EC2 concentration, close to the estimated *C*_e_, as discussed above, produces a hybrid architecture with beaded thin fibers in combination with clusters of particles. In this regard, Tang et al.,^37^ who studied the electrospinnability of poly(vinyl alcohol) (PVA) nanofibers, determined that the critical concentration to spin bead-free nanofibers was ∼2.5 times greater than the entanglement molecular weight concentration, *C*_e_. In this case, this proportion provides a critical concentration of 8.5 wt % to achieve totally bead-free nanofibers, which is in agreement with the experimental observations. In addition, to investigate the electrospinnability of EC_2_ solutions, the relationship between the average fiber diameter and the specific viscosity is displayed in Figure 2. The data points of the solutions that could be clearly electrospun into beaded or bead-free fibers are shown in the figure, and an exponential relationship was observed between the fiber diameter and the specific viscosity.

#### 3.1.2 Effect of Ethylcellulose Molecular Weight on Electrospinning

**Figure 3:**
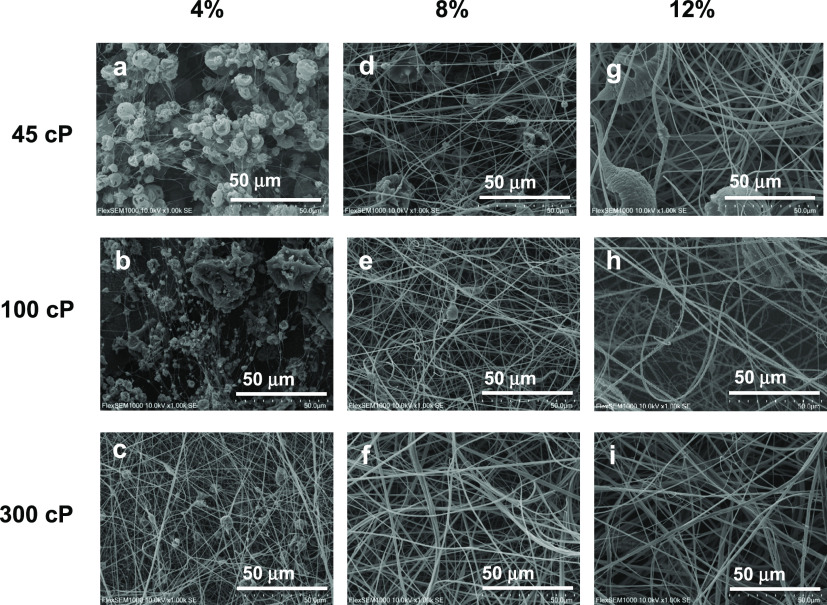
Effect of ethylcellulose molecular weights (45, 100, and 300 cP) on the morphology of electrospun nanostructures: 4 wt % (a–c), 8 wt % (d–f), and 12 wt % (g–i).

**Figure 4:**
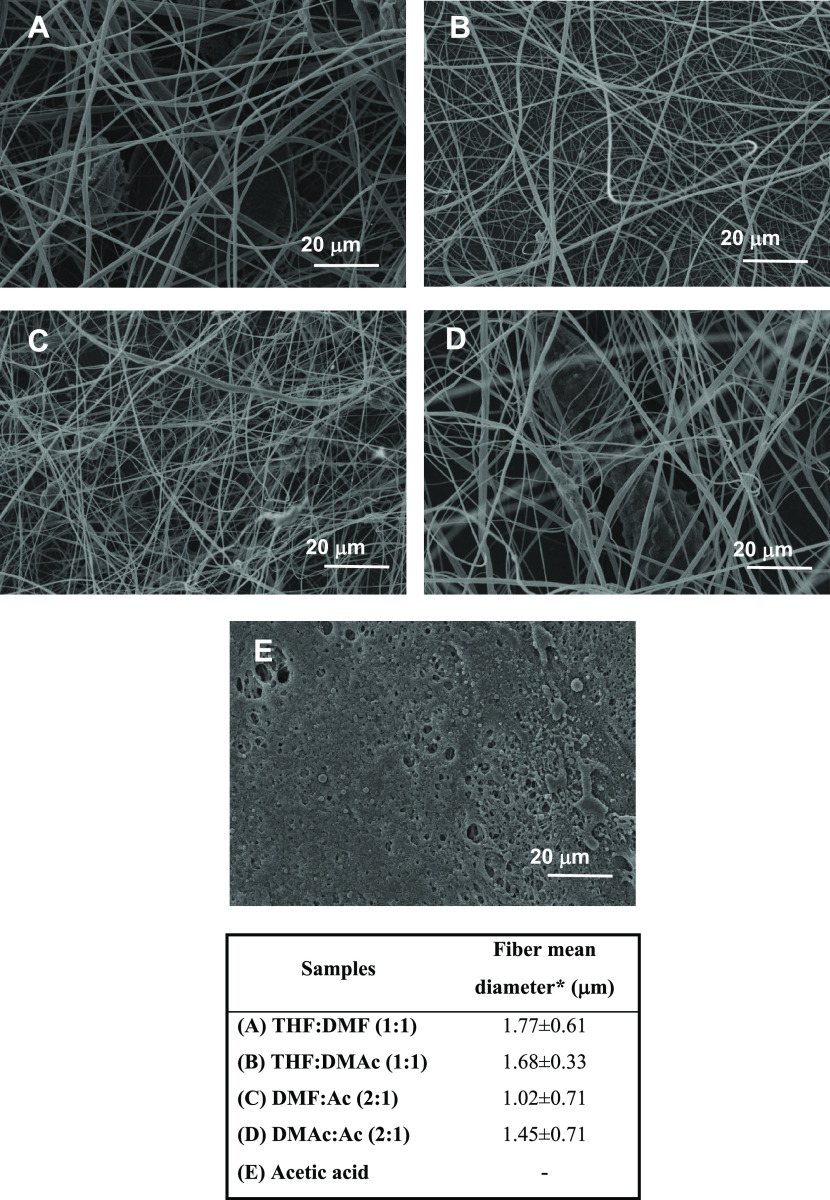
Effect of solvent systems on the morphology of electrospun nanostructures. SEM micrographs obtained from 10 wt % ethylcellulose (EC_2_) solutions in (A) THF/DMF, (B) THF/DMAc, (C) DMF/acetone, (D) DMAc/acetone, and (E) acetic acid. The table collects the average mean fiber diameters obtained from these pictures.

As has been noted previously, the viscosity of the spinning solution is closely related to the morphology of the fibers obtained, one of the determining parameters directly influencing the viscosity of the solution being the polymer molecular weight (*M*_w_). Viscosity influences the concentration regimes; therefore, the choice of the molecular weight determines, together with other parameters, the morphological changes. To study the effects of the molecular weight, solutions of ethylcellulose with different *M*_w_ values, i.e., 45 cP (EC_1_), 100 cP (EC_2_), and 300 cP (EC_3_), were prepared to attain concentrations of 4, 8, and 12 wt %, respectively. To the best of our knowledge, the influence of the ethylcellulose molecular weight on the morphology of electrospun nanostructures has never been reported. As can be observed in Figure 3, EC with higher molecular weight (EC_3_) produced defect-free fibers at a concentration of 8 wt %, and beaded fibers at 4 wt %. However, for EC with a lower molecular weight (EC_1_), it was necessary to increase the concentration of the solution until 12 wt % to achieve bead-free electrospun fiber mats and even some embedded particles are eventually detected (see Figure 4G). In addition, for the same concentrations, the higher EC molecular weight, the larger average fiber diameter was. According to these results, uniform electrospun fiber mats were achieved above a minimum critical concentration, which depends on the EC molecular weight. This certain EC critical concentration for a 45 cP EC was above 12 wt %, whereas for the 300 cP EC, it was below 8 wt %. Or, from another perspective, to obtain morphological appearance and fiber diameters similar to those of the nanostructures obtained with the spinning solution of high molecular weight (EC_3_, 300 cP) at a concentration of 4 wt %, the concentration of the EC_1_ (45 cP; *M*_w_ ∼ 2 times lower) solution had to be increased to 8 wt %. The critical entanglement concentration (*C*_e_) values determined from specific viscosity vs. EC concentration plots, for each of the molecular weights, were estimated to be 4.3, 3.4, and 2.7 wt % for EC_1_, EC_2_, and EC_3_, respectively (Figure S3 in the Supporting Information). According to these *C*_e_ values, and considering the ∼2.5 proportionality previously discussed, for the lowest molecular weight, an EC concentration of around 11 wt % would be needed to obtain bead-free nanofibers; however, if the EC molecular weight was increased to 100 and 300 cP, the concentration required would be reduced to 8.5 and 6.5 wt %, respectively, which is approximately in agreement with the experimental morphological observations (Figure 3). Therefore, increasing the molecular weight in the EC solutions increases the molecular entanglement of the biopolymer and improves the electrospinnability, which leads to larger fiber diameters and more homogeneous electrospun defect-free nanofiber mats.

#### 3.1.3 Effect of Solvent Systems on Electrospinning

To determine the optimal solvent systems for EC electrospinning, several EC_2_ spinning solutions at 10 wt % concentration in 1:1 THF/DMF, 1:1 THF/DMAc, 1:2 acetone/DMF, 1:2 acetone/DMAc, and acetic acid were electrospun. Figure 4 shows the SEM micrographs of the EC electrospun nanofiber mats in these mixed solvents and their average fiber diameters. As can be observed, at this EC2 concentration, all solvent systems studied were found to produce a sufficient quantity of bead-free fibers to form homogeneous electrospun fiber mats, with the exception of acetic acid (AA) that does not allow fibers to be obtained but a compact morphology instead (see Figure 4E). This fact is probably related to its relatively low dielectric constant and dipole moment compared to the other mixed solvents, combined with its higher boiling point (>100 °C) (see Supporting Information Table S1). In this case, the charged jets could not be dried enough before being collected on a grounded target. This result is in very good accordance with the literature findings, where AA was reported to be the less favorable solvent for cellulose acetate, yielding no uniform electrospun mats.^38^ Comparing the binary solvent systems, both the THF-based and the acetone-based binary solvents are adequate to determine the effect of solvents on the morphology of electrospun fiber mats. According to the physicochemical properties of solvents (see Table S1), if THF was replaced by acetone in the binary system, the dielectric constant and dipole moment will be remarkably increased, while the density, surface tension, viscosity, and boiling point will be decreased. As may be observed in Figure 4, uniform nanofiber mats were collected when using all of the binary solvent systems studied; however, it was found that the average fiber diameters decreased when the dielectric constant and dipole moment of the solvents increased and the viscosity and boiling point of the solvents decreased, i.e., acetone-based binary solvents. Chuangchote et al. found a similar correlation between the dielectric constant and dipole moment of the solvent and resulting fiber diameters for the electrospinning of poly(vinyl pyrrolidone) in different alcohol solutions (methanol, ethanol, and 2-propanol),^39^ whereas Lee et al. found that the solvent dielectric constant is one of the key properties in the electrospinning process.^40^ Solvents with higher dielectric constants lead to greater Coulombic repulsion forces, which are responsible for the stretching of the charged jet, and electrostatic forces, which are responsible for carrying the charged jet to the collector. In this sense, the dielectric constant and dipole moment of the binary solvent systems investigated played an influential role in the morphological features of EC nanofibers. However, despite this, the differences found in the diameter of fibers cannot be considered very relevant. In general, the appearance of the electrospun fiber web obtained with these binary solvent systems was somewhat heterogeneous, exhibiting some beaded fibers and/or eventual embedded particles, except for the THF/DMAc solvent system.

### 3.2 EC Electrospun Nanofiber Dispersions in Castor Oil

To study the ability of different EC electrospun structures obtained to form physically stable dispersions, the micro- and nanostructures were mixed with castor oil at 20 wt % using the methodology described in the Experimental Section. The structures were selected from EC spinning solutions at concentrations ranging from 4 to 12 wt % and the three molecular weights (EC_1_, EC_2_, and EC_3_). Nanostructures obtained with spinning solutions below 4 wt %, i.e., essentially not well-developed nanofiber webs, provided physically unstable dispersions.

#### 3.2.1 Rheological Properties

**Figure 5:**
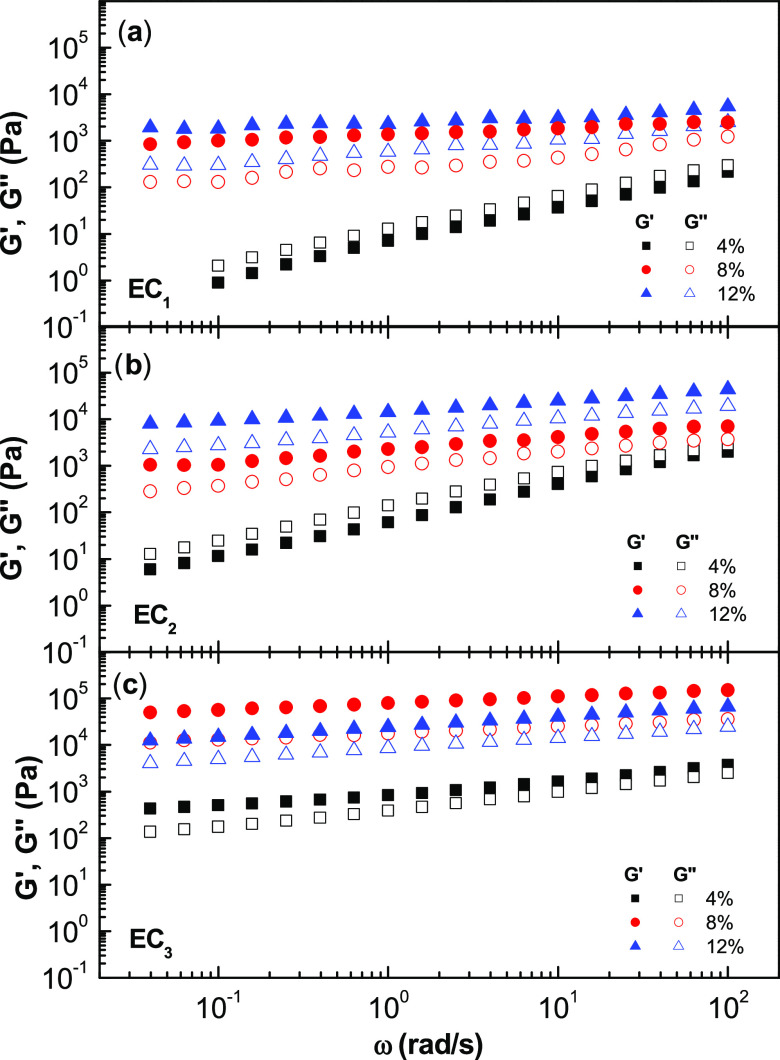
Evolution of the storage, *G*′ (filled symbols), and loss, *G*″ (open symbols), moduli with frequency for dispersions of ethylcellulose electrospun nanofibrous webs in castor oil, as a function of the concentration in the spinning solution and the ethylcellulose molecular weight of the biopolymer: (a) 45 cP, (b) 100 cP, and (c) 300 cP.

**Figure 6:**
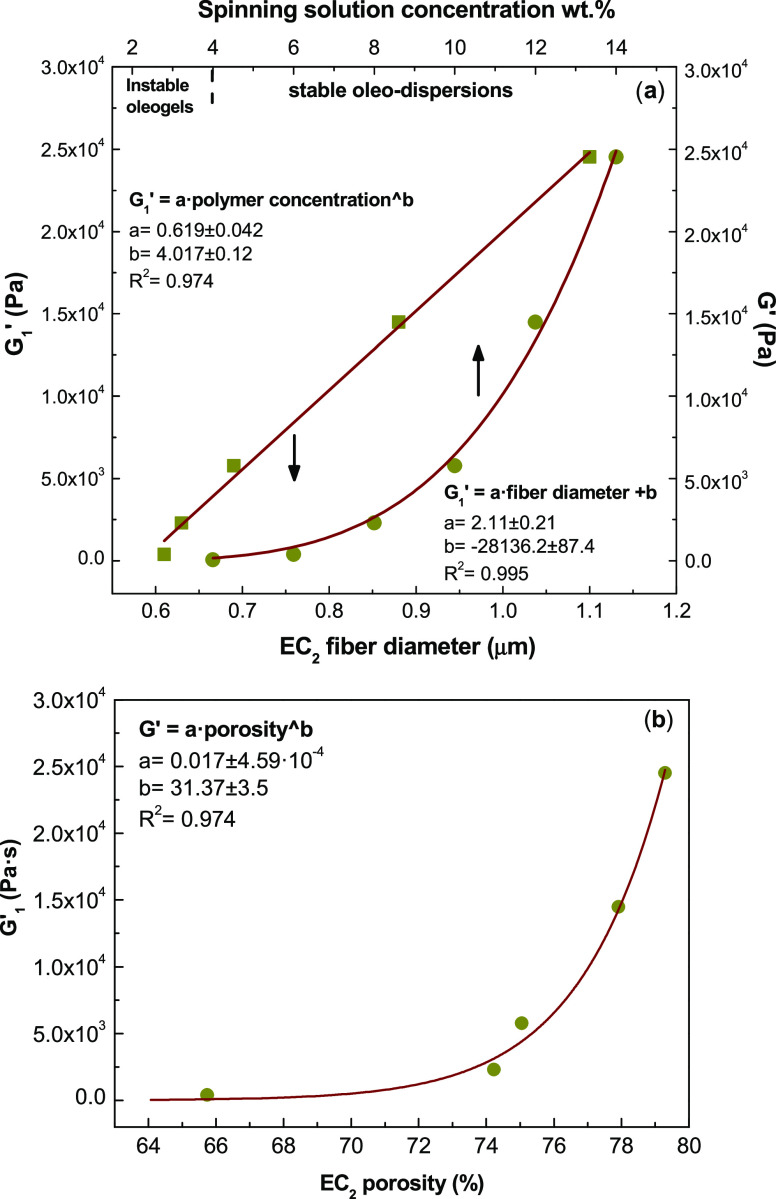
Evolution of the storage modulus, at 1 rad/s (*G*_1_′), as a function of (a) the spinning solution concentration and average fiber diameter and (b) nanostructure porosity for dispersions of ethylcellulose (EC_2_) electrospun nanofibrous webs in castor oil.

**Figure 7:**
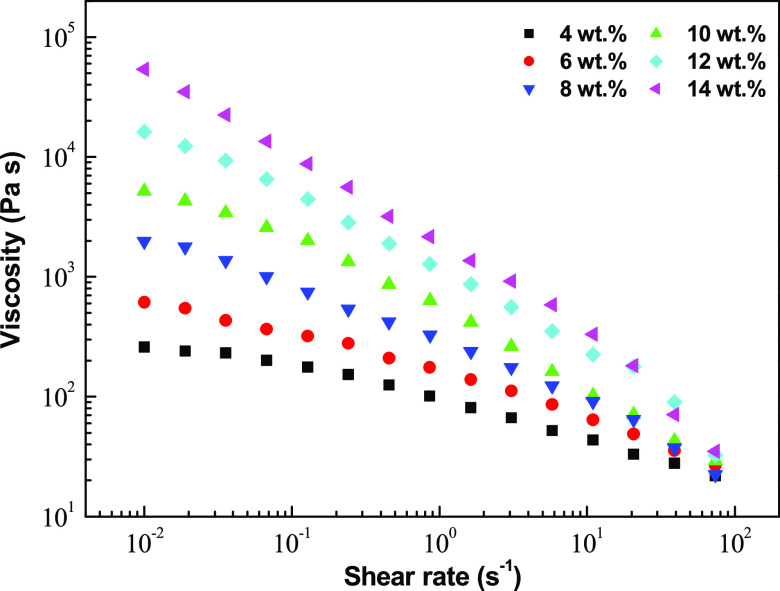
Evolution of the apparent viscosity with shear rates for dispersions formulated with ethylcellulose nanostructures obtained from spinning solutions having different EC_2_ concentrations.

The evolution of the storage (*G*′) and loss (*G*″) moduli with frequency for dispersions formulated with EC nanostructures is shown in Figure 5. In most cases, the mechanical spectra of all samples show a typical weak gel-like response with *G*′ values higher than those of *G*″. As can be seen, the evolution of both moduli with frequency depends on the EC molecular weight and, especially, on the spinning polymer concentration. In addition, dispersions formulated with nanostructures obtained from solutions containing 4 wt % EC with lower molecular weights (EC_1_ and EC_2_) display a viscoelastic fluid response (tan δ = *G*″/*G*′> 1). *G*′ and *G*″ values increased with the spinning solution concentration and EC molecular weight. Nevertheless, an exception was found for the dispersion of EC_3_ (300 cP) nanofibrous web obtained from the solution with the highest concentration, where a decrease in the viscoelastic functions was observed. This behavior could be attributed to the strong H-bonds among the long chains of EC_3_ molecules that inheritably keep the molecules in their own conformation, hindering the entry of oil molecules into the EC_3_ network during the dispersion manufacture. Figure 6 illustrates the influence of nanofiber morphology on the linear viscoelastic response of the resulting dispersions formulated with EC_2_. Figure 6a displays the evolution of the storage modulus obtained from the mechanical spectrum at 1 rad/s, *G*_1_′, as a function of the spinning polymer concentration and fiber diameter. As can be observed, *G*_1_′ increases with both spinning polymer concentration and fiber diameter and evolves potentially and linearly, respectively, in the experimental ranges studied. The influence of the porosity of electrospun nanostructures on the viscoelastic behavior of derived dispersions is also illustrated in Figure 6b. As can be observed, G_1_′ increases potentially with the porosity, which could be a consequence of a spongier nanostructure with a higher effective specific surface area to retain the oil. These results could be related to the wetting behavior of the electrospun EC nanostructures. As is known, the capacity of some modified cellulose-based products to retain or absorb oils is closely related to the physicochemical properties of the surface.^41^ The results could also be corroborated through the physical appearance of the different oleo-dispersions (see Figure S4 of the Supporting Information). Thus, these empirical correlations allow estimating the physical stability and linear viscoelastic response of the oleo-dispersions from the nanoarchitecture (fiber size and porosity) of electrospun fiber webs and, indirectly, from the intrinsic properties of EC solutions. The viscous flow behavior of EC_2_ electrospun nanofiber dispersions in castor oil was also studied, and the evolution of the apparent viscosity with shear rate is shown in Figure 7 as a function of the concentration of the spinning solution. The viscosity of the EC electrospun nanofiber dispersions increases monotonically with the spinning solution concentration (i.e., with fiber diameter) by 2.5 orders of magnitude at low shear rates when the concentration was modified from 4 to 14 wt %. All of the dispersions exhibited a shear-thinning flow behavior that is more pronounced as the concentration of the spinning solution increases. The resistance of the entangled fiber network to flow is relatively strong at small shear rates and becomes weaker at high shear rates due to a reorientation and/or breakdown of fibers. Dispersions with higher spinning solution concentrations (12 and 14 wt %) exhibited fracture in the sample at high shear rates (>30 s^–1^).

**Figure 8:**
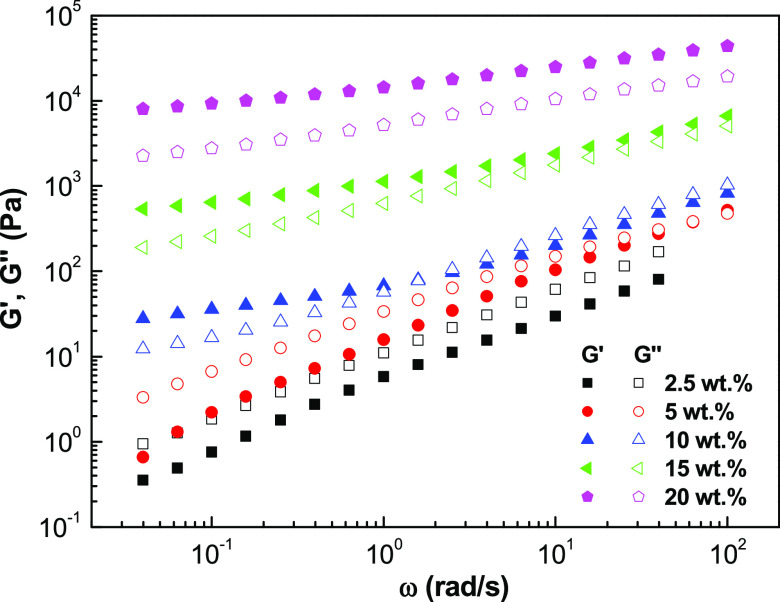
Evolution of the storage, *G*′ (filled symbols), and loss, *G*″ (open symbols), moduli with frequency for dispersions of ethylcellulose (EC_2_) electrospun nanofibrous webs in castor oil as a function of the nanofiber concentration.

The impact of the concentration of dispersed EC electrospun nanofibers on SAOS tests was also investigated. Figure 8 displays the evolution of the viscoelastic functions of EC_2_ electrospun nanofiber dispersions in castor oil as a function of concentration. As can be seen, for lower thickener concentrations (2.5–10 wt %), liquid-like viscoelastic responses were obtained, where both *G*′ and *G*″ markedly increased with frequency and *G*″ values lay above those of *G*′ until reaching a crossover at a certain frequency, which decreased with the nanofiber concentration. The reciprocal of the crossover frequency allows the terminal relaxation time to be estimated, the increase in the terminal relaxation time being a clear indication of the progressive relevance of the elastic response as nanofiber concentration was increased. On the other hand, dispersions with higher EC_2_ electrospun nanofiber concentrations (15 and 20 wt %) evinced weak gel-like viscoelastic properties with a predominance of the storage modulus (*G*′) over the loss modulus (*G*″) in the frequency range studied and much lower frequency dependence of both moduli. This change in rheological response could be attributed to a higher degree of interactions among adjacent EC fibers.^42^

**Figure 9:**
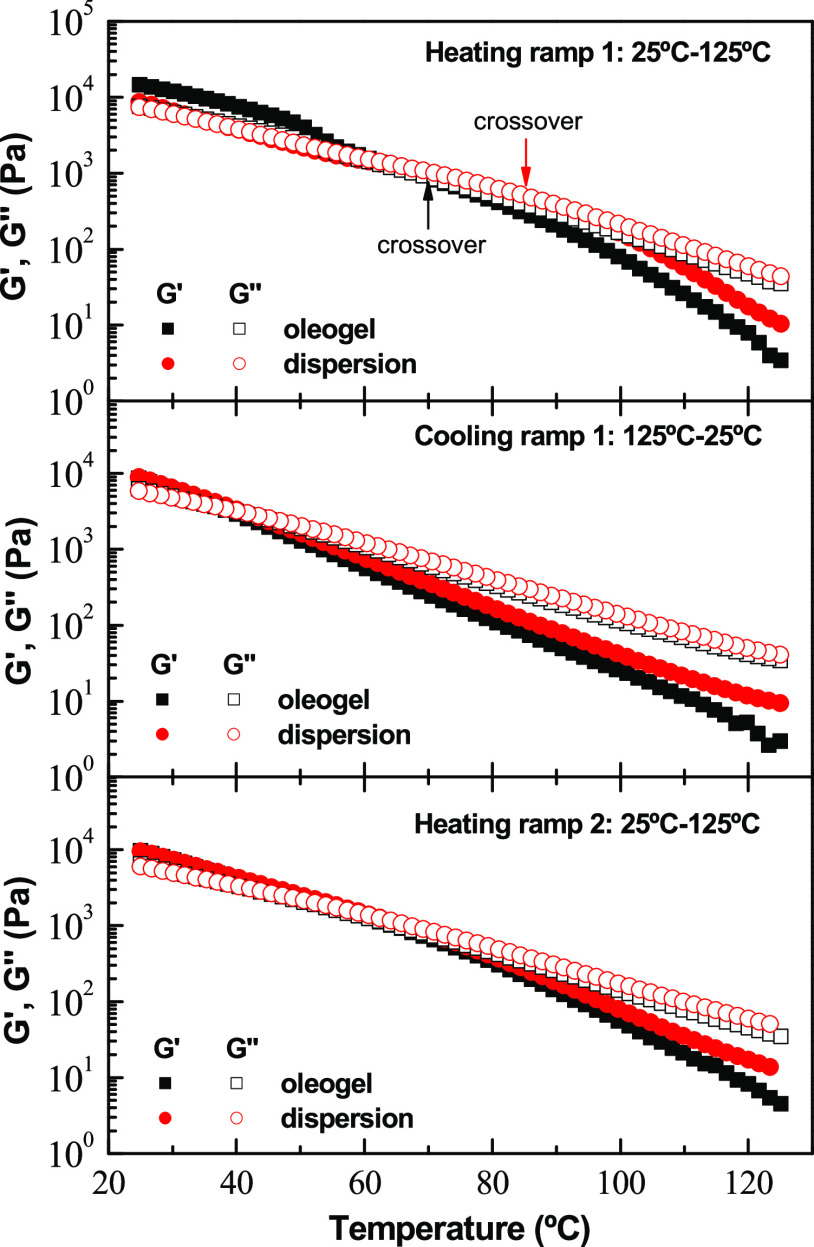
Evolution of the storage, *G*′ (filled symbols), and loss, *G*″ (open symbols), moduli with temperature for a selected dispersion of ethylcellulose (EC_2_) electrospun nanofibrous web in castor oil when applying a three-step heating–cooling–heating cycle as compared with that shown for the oleogel prepared by thermogelation (EC_2_ concentration: 15 wt %).

Moreover, temperature sweep tests in the linear viscoelastic regime were performed on a selected EC_2_ electrospun nanofiber dispersion, at 15 wt %, and compared with those performed on the respective EC_2_ oleogel prepared by applying the typical thermogelation mechanism, i.e., by solubilizing EC_2_ at 140 °C and cooling down to room temperature. These temperature sweep tests aim to analyze more deeply the thermorheological response of oleogel-like samples processed with different approaches. The test was carried out as a three-cycle experiment, which includes one heating to 125 °C, cooling down to 25 °C, and an additional second heating stage to 125 °C, to assess the thermoreversibility of the microstructural networks. Figure 9 displays the evolution of the linear viscoelastic moduli (*G*′ and *G*″) with temperature for both the EC_2_ electrospun nanofiber dispersion and the equivalent oleogel. As can be observed, in the first heating, at low temperatures, the storage modulus (*G*′) is higher than the loss modulus (*G*″) and both moduli gradually decreased when the temperature increased until a crossover was reached, and then *G*″ became higher than *G*′. The decrease in the linear viscoelastic moduli reflects a rearrangement and partial disruption of the entanglement network by heating, mainly due to a weakening of physical interactions, mostly hydrogen bonds.^43^ This decrease is more pronounced for oleogel than for nanofiber dispersion. This fact could be attributed to the sol–gel transition of the network in the sample.^44^ Interestingly, a clear influence of the processing protocol on the values of the crossover temperature can be observed. EC_2_ electrospun nanofiber dispersion displayed a crossover temperature of about 15 °C higher than oleogel. Upon cooling and second heating, thermal reversibility was observed in both samples, with values of the linear viscoelastic moduli slightly higher for the dispersion and similar crossover points for both samples, which otherwise were shifted to lower temperatures. Therefore, according to these results, EC_2_ electrospun nanofiber dispersions withstand medium–high temperatures similarly or better than the oleogel and displayed good thermal reversibility. The faster weakening of the conventional oleogel structure during the first heating reflects a more fragile nature of this network, which can disrupt more easily than the percolation network of electrospun nanofibers. After the first heating cycle, thermally induced rearrangements of both networks are very similar.

#### 3.2.2 Tribological Properties

Finally, to explore one possible industrial application of these samples (nanofiber dispersion and conventional oleogel) as environment-friendly semisolid lubricant formulations, the friction coefficient was assessed in a ball-on-plate steel–steel tribological contact. Table 1 collects the stationary values of the friction coefficient and the corresponding average diameter of wear scars generated during the friction experiments. Both samples showed a satisfactory tribological response. Thus, the friction coefficient values were comparable to those obtained under similar conditions with a multipurpose lithium semisolid lubricant,^45^ as well as with other gel-like dispersions based on synthetic polymers previously proposed as bio-based lubricants.^46^ Interestingly, the EC_2_ electrospun nanofiber dispersion used as a lubricant provided an extraordinarily low friction coefficient value and smaller wear scars than that obtained with the conventional oleogel. This fact could be attributed to the different entanglement networks achieved by both methods. In this sense, the electrospun nanofiber percolation network seems to have a greater ability to release the lubricating oil into the tribological contact, thereby improving the frictional and wear behavior.

**Table 1:** Values of the Friction Coefficient and Average Diameter of Wear Scars Obtained with Both a Selected Dispersion of Ethylcellulose (EC_2_) Electrospun Nanofibrous Web in Castor Oil and the Equivalent Oleogel Prepared by Thermogelation When Acting as Lubricants in a Tribological Contact (EC_2_ Concentration: 15 wt %)

sample	friction coefficient	wear scar diameter (μm)
ethylcellulose electrospun nanofiber dispersion	0.075 ± 2.5 × 10^–3^	359.7 ± 18.3
oleogel (from thermogelation)	0.110 ± 1.4 × 10^–3^	433.1 ± 24.1

## 4 Conclusions

Nanofibrous webs of ethylcellulose (EC) were successfully produced via electrospinning and validated for structuring castor oil. The morphology of EC nanostructures can be modulated by modifying the properties of the spinning solution, including EC concentration and molecular weight, and solvent. Particle networks and/or hybrid nanostructures comprised of thin fibers in combination with clusters of particles were collected from solutions with EC concentrations below or around the critical entanglement concentration (*C*_e_), while defect-free nanofibers were produced when the concentration was increased to about 2.5 times *C*_e_ regardless of the EC molecular weight. An increase in both EC molecular weight and concentration improves electrospinnability resulting in a higher number of entanglements in the nanofibrous web and larger average fiber diameters. The physicochemical properties of binary solvent systems, especially dielectric constant and dipole moment, play an important role in the morphology of EC nanofiber webs. Among all of the solvent systems employed, THF/DMAc provides the most homogeneous nanofiber mats and the best electrospinning performance. EC nanofibrous webs obtained from solutions above *C*_e_ are able to form physically stable gel-like dispersions by simply mixing them in castor oil, at room temperature, for nanofiber concentrations above 15 wt %. Instead, liquid-like viscoelastic dispersions were obtained at nanofiber concentrations of 2.5–10 wt %. In addition, the morphology of the nanoarchitectures generated exerted a great impact on the rheological behavior of the resulting dispersions. Typical gel-like behavior was exhibited by dispersions of homogeneous and defect-free nanofibrous webs, while structures based on combinations of beaded fibers and particles give rise to predominantly liquid-like rheological responses. EC electrospun nanofiber dispersions in castor oil display very good thermal reversibility and better thermorheological and tribological behavior than conventional EC oleogels prepared by thermogelation. Overall, the electrospinning of ethylcellulose nanofibrous webs can be proposed as an alternative approach for structuring vegetable oils, which may have great importance in a diverse range of applications in fields such as lubricants, food, and pharmaceuticals.

## Supporting Information Available

Manufacturing process of EC gel-like dispersions (Scheme S1); viscous flow curves of ethylcellulose (EC2) solutions in 1:1 THF/DMAc as a function of concentration (Figure S1); specific viscosity versus concentration plot for ethylcellulose (EC2) solutions in 1:1 THF/DMAc (Figure S2); specific viscosity versus concentration plots for ethylcellulose solutions in 1:1 THF/DMAc for different ethylcellulose molecular weights (Figure S3); physical appearance of oleo-dispersions formulated with ethylcellulose nanostructures obtained from spinning solutions having different EC2 concentrations: (a) 4 wt %, (b) 6 wt %, (c) 8 wt %, (d) 10 wt %, (e) 12 wt %, and (f) 14 wt % (Figure S4); and physical properties of the solvents used to prepare binary solvent systems for electrospinning of ethylcellulose (Table S1) (PDF)
