# Supplementary material for: Developing Electrospun Ethylcellulose Nanofibrous Webs: An Alternative Approach for Structuring Castor Oil
Source: ACS Appl Polym Mater. 2022 Sep 8;4(10):7217–27. doi: 10.1021/acsapm.2c01090 (PMC10552660; doi:10.1021/acsapm.2c01090)
Supplement: Supplementary file 1 — ap2c01090_si_001.pdf [file ap2c01090_si_001.pdf]

Supporting Information

Developing electrospun ethylcellulose nanofibrous webs: an alternative approach for structuring castor oil

M. Borrego, J.E. Martín-Alfonso*, C. Valencia, María del Carmen Sánchez Carrillo, J.M. Franco

Department of Chemical Engineering and Materials Science, Campus de "El Carmen", University of Huelva, Chemical Product and Process Technology Research Center (Pro^{2}TecS) 21071 Huelva, Spain.

*Email: jose.martin@diq.uhu.es

Supporting Information contents

Number of pages: 7

Number of Schemes: 1

Number of figures: 4

Number of tables: 1

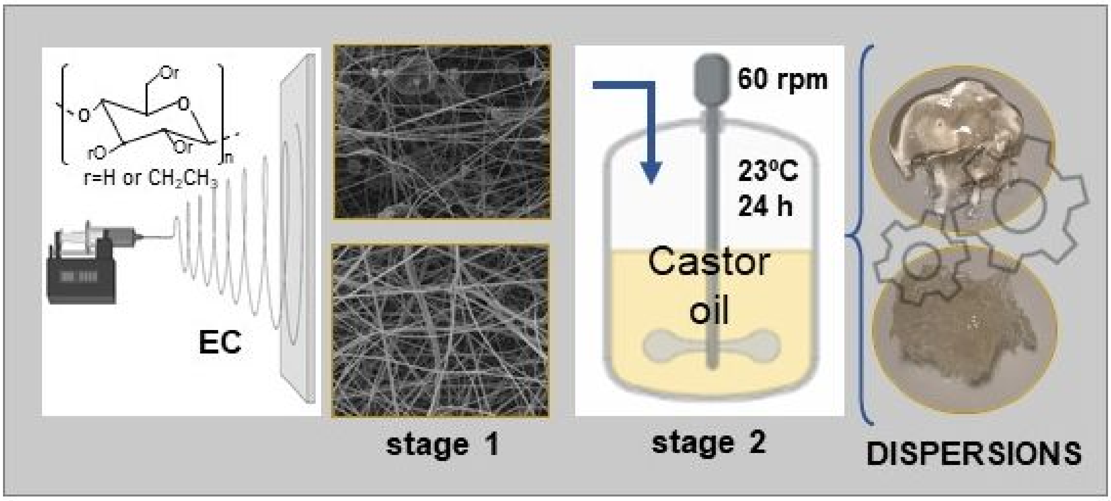

Scheme S1. Manufacturing process of EC gel-like dispersions.

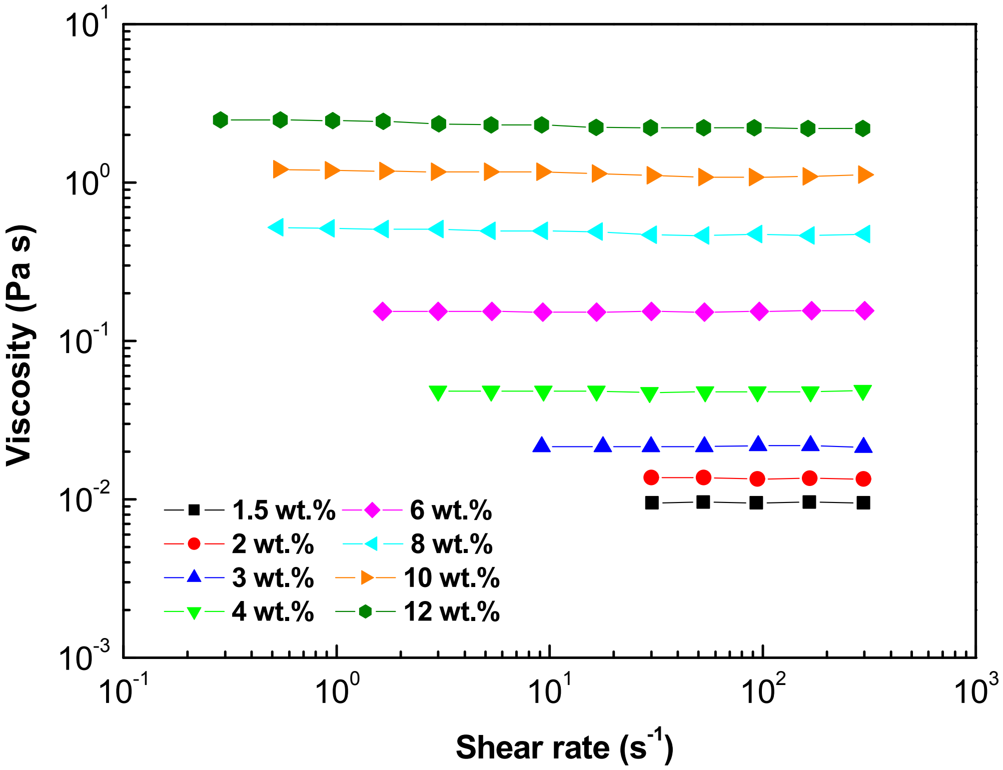

Figure S1. Viscous flow curves of ethylcellulose (EC2) solutions in 1:1 THF:DMAc as function of concentration.

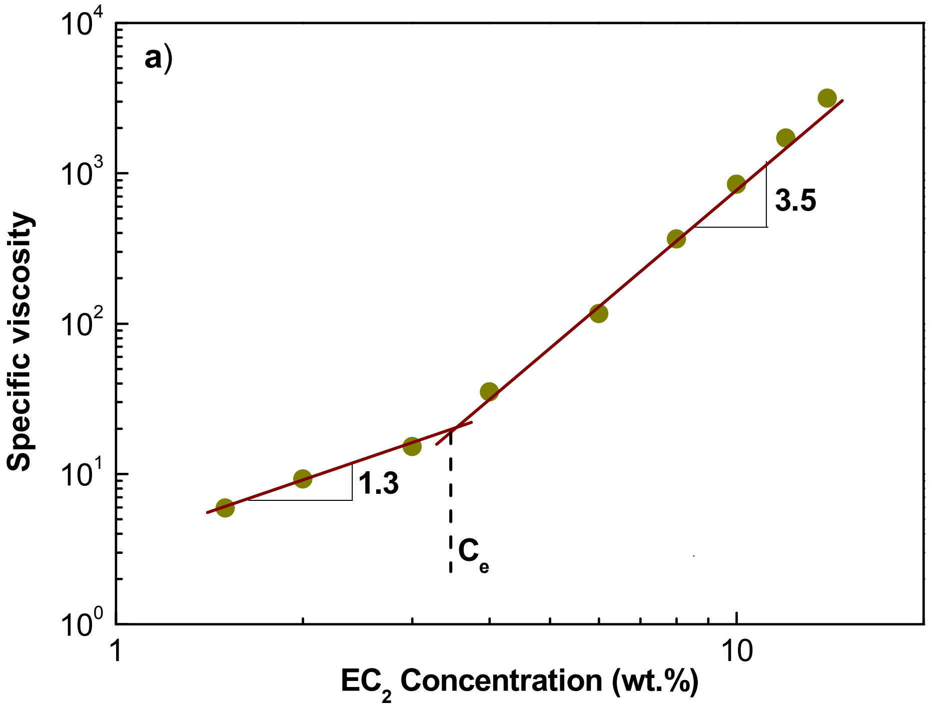

Figure S2. Specific viscosity versus concentration plot for ethylcellulose (EC2) solutions in 1:1 THF:DMAc.

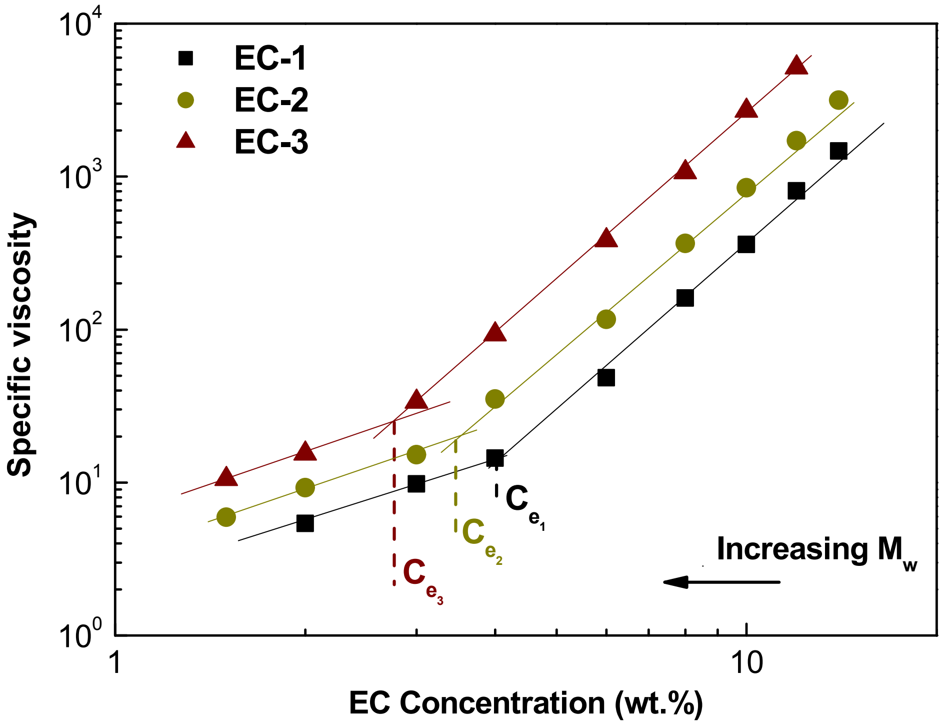

Figure S3. Specific viscosity versus concentration plots for ethylcellulose solutions in 1:1 THF:DMAc, for different ethylcellulose molecular weights.

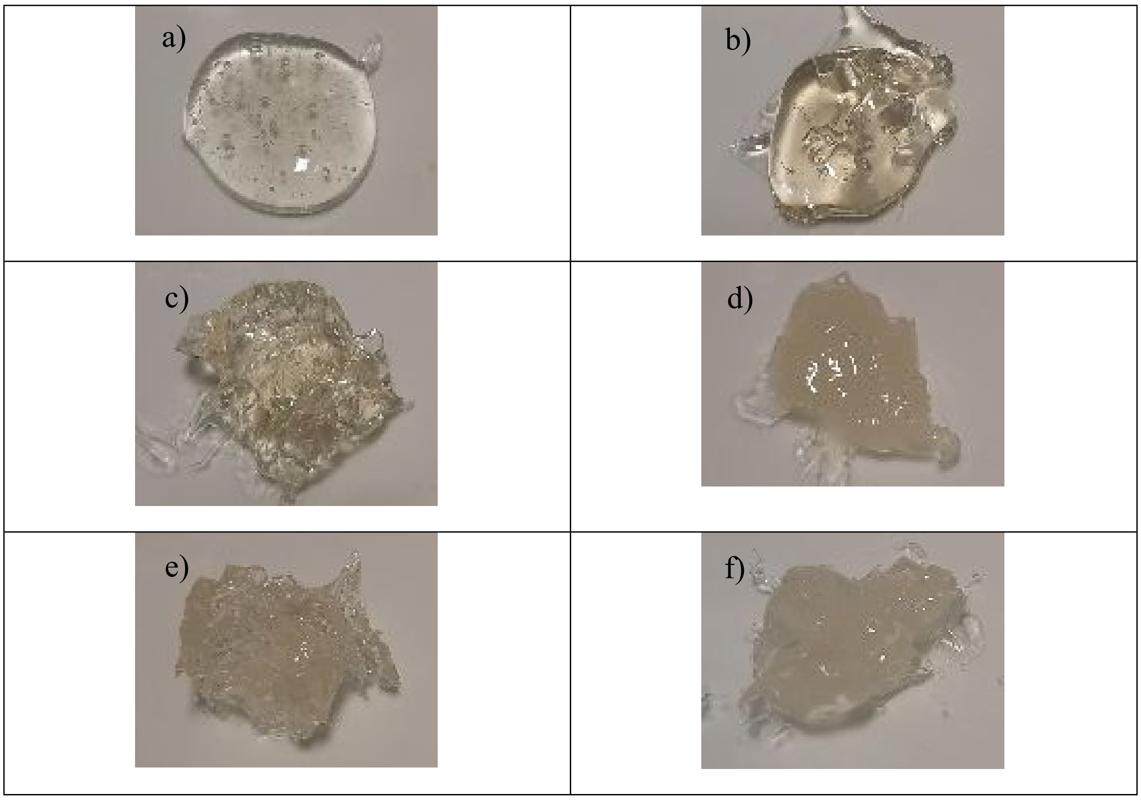

Figure S4. Physical appearance of oleo-dispersions formulated with ethylcellulose nanostructures obtained from spinning solutions having different EC_2 concentrations: a) 4 wt.%; b) 6 wt.%; c) 8 wt.%; d) 10 wt.%; e) 12 wt.% and f) 14 wt.%.

Table S1. Physical properties of the solvents used to prepare binary solvent systems for electrospinning of ethylcellulose.

Solvents	Boiling point (ºC)	Viscosity (mPa S)	Surface tension (mN/m)	Density (g/cm^3)	Dipole moment (D)	Dielectric constant
**THF**	66	0.48	26.4	0.875	1.75	7.6
**DMF**	152	0.92	37.1	0.945	3.86	36.7
**DMAc**	166	0.94	36.7	0.937	3.72	37.8
**Acetone**	56	0.32	23.1	0.788	2.85	20.7
**Acetic acid**	118	1.10	27.4	1.049	1.68	6.15
